# Haemoglobin and haematocrit: the threefold conversion is also non valid for assessing anaemia in *Plasmodium vivax *malaria-endemic settings

**DOI:** 10.1186/1475-2875-6-166

**Published:** 2007-12-17

**Authors:** Alfonso J Rodríguez-Morales, Elia Sánchez, Melissa Arria, Miguel Vargas, Carmelina Piccolo, Rosa Colina, Carlos Franco-Paredes

**Affiliations:** 1 Instituto Experimental José Witremundo Torrealba, Universidad de Los Andes, Trujillo 3102, Venezuela; 2 Infectious Diseases Service, Hospital Santos Anibal Dominicci, Carúpano, Sucre, Venezuela; 3 Postgraduate Course on Genetics, Venezuelan Institute for Scientific Research (IVIC), Caracas, Venezuela; 4 Division of Infectious Diseases, Emory University School of Medicine, TravelWell Clinic 550 Peachtree Street, MOT CLH, Atlanta GA, 30308, USA; 5 Hospital Infantil de México, Federico Gómez, México DF, México

## Abstract

It has been recently reported that the standard threefold conversion from haematocrit to haemoglobin underestimates the prevalence of anaemia and low levels of haemoglobin in children living in areas endemic for *Plasmodium falciparum *malaria. The data presented herein describes the experience in a malaria-endemic zone in northeastern Venezuela (state of Sucre), where a similar bias between haematocrit and haemoglobin in patients with *Plasmodium vivax *infection was found. In summary, the relationship between haematocrit and haemoglobin needs to be specifically evaluated according to each particular region or epidemiological setting.

## Background

Carneiro *et al *[1] recently reported that the standard threefold conversion from haematocrit to haemoglobin underestimates the prevalence of anaemia and low levels of haemoglobin in children under five years of age in malaria endemic settings.

In agreement with Carneiro *et al *findings, demonstrating that the usual threefold conversion represents a significant bias when haemoglobin (Hb) is estimated based on haematocrit (Hct) values in children with malaria, the present report describes the experience in a vivax malaria-endemic zone in northeastern Venezuela. It has been a widely accepted assumption that this conversion could be used as an alternative measurement to haemoglobin in malaria studies [2]. This is particularly important in African settings where *Plasmodium falciparum *malaria is endemic, but the corresponding haemoglobin levels cannot be derived with an acceptable accuracy using the value three as a conversion factor [2]. As a consequence, the relationship between haematocrit and haemoglobin needs to be specifically evaluated according to each particular region or epidemiological setting. In order to illustrate this important issue, the experience in a malaria-endemic zone in northeastern Venezuela (state of Sucre) is described [3,4], showing a similar bias between haematocrit and haemoglobin in patients with *Plasmodium vivax *infection.

## Methods and results

Data from one study that have measured haemoglobin and haematocrit was used to assess the reliability of the standard threefold conversion factor. Finger-prick blood samples were collected for determination of anaemia status from 120 patients with malaria aged 4–89 years-old from a prospective survey carried out between 2000 and 2002 in Carupano, Sucre, Venezuela [5]. In this study, haemoglobin concentration was assessed by haemophotometry and haematocrit was assessed by centrifugation using standard procedures for microhaematocrit tubes and centrifuge (i.e. 10 minutes at a fixed speed of 11,000 rpm).

### Statistical methods

As described by Carneiro *et al *[1], the measurement of observed and estimated haemoglobin using the method of plotting the difference between the measures against the mean of the two measures, as reported by Bland & Altman [6], is compared. Then, a comparison between haemoglobin (g/dl) and haematocrit (%) divided by a factor of three was done to be able to compare the measurements on approximately the same scale ("grams of haemoglobin per dl"). The difference between the haemoglobin and haematocrit/3 measurements (i.e. Hb - Hct/3) and the mean of the two measurements (i.e. (Hb + Hct/3)/2 – now called average to avoid confusion with mean difference), were calculated for each individual. Linear regression analyses were used to define the relationship between the mean difference and the average of the two measures [6], adjusting for *a priori *covariates of age and sex. The differences between both measures were also tested in terms of means comparison using the Student's *t *test for one-sample, for independent-samples and paired-samples.

**Figure 1:**
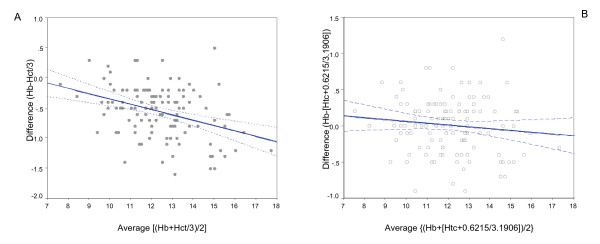
Scatter-plots of difference against average of haemoglobin and haematocrit/3 (A), as well after correction using (haematocrit+0.6215)/3.1906 (B). Scatter-plots of difference against average of haemoglobin and haematocrit/3 for paired measurements, and between average of haemoglobin and (haematocrit+0.6215)/3.1906. The line of best fit (blue) indicates the trend towards greater differences at higher haemoglobin values (significantly lower after correction). Both axes are in "grams of haemoglobin/dl".

Haemoglobin measurements were lower than haematocrit/3 in this study in 92.5% (111/120), with a linear trend in the relationship so that more negative differences were seen with increasing haemoglobin levels (*r*^2 ^= 0.1379, F = 18.87, P < 0.0001) (Figure 1). Logarithmic transformations of the means and differences as suggested by Bland & Altman [7] did not improve this relationship.

The mean difference between haemoglobin and haematocrit/3 values was -0.6 (± 0.4, range -1.6 to 0.5). The differences between both measures in terms of means comparison for Student's *t *tests were also significantly different (Table 1). When the haematocrit was calculated using the haemoglobin (haemoglobin × 3), similar significant (P < 0.05) differences were evidenced between the observed and estimated haematocrit. These differences not showed a statistically significant variation in regard to the age neither with the sex (P > 0.05).

**Table 1:** Mean difference comparisons between observed haemoglobin and estimated haemoglobin (Haematocrit/3, expressed as "g of haemoglobin/dl")

	Observed Haemoglobin (n = 120)	Estimated Haemoglobin (Haematocrit/3) (n = 120)
Mean	12.06	12.62
Standard deviation	1.71	1.87
Standard error	0.16	0.17

*One-sample test*		
t	77.23	73.94
Mean difference	12.06	12.62
95% CI of the difference	11.75 to 12.37	12.28 to 12.95
P	<0.001	<0.001

*Independent-sample test*		
t	-	-2.399
Mean difference	-	-0.555
95% CI of the difference	-	-1.017 to -9.9328E-02
P	-	0.017

*Paired-sample test*		
t	-	-14.284
Mean difference	-	-0.555
95% CI of the difference	-	-0.6319 to -0.4781
P	-	<0.001

**Figure 2:**
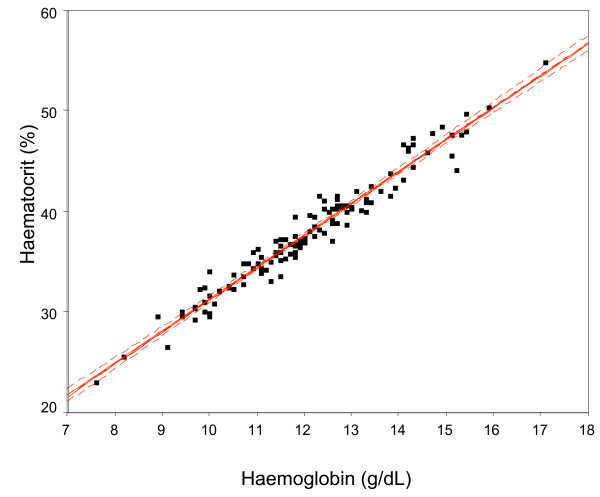
Scatter-plot of the observed haemoglobin and observed haematocrit relations. The line of best fit (red) indicates a trend towards higher haemoglobin and haematocrit values, giving the following Equation 1: Estimated Haemoglobin = (Haematocrit + 0.6215)/3.1906.

Then, the relation between observed haematocrit and observed haemoglobin was modelated (Figure 2)(y = 3.1906× - 0.6215). The final regression model gave the following relationship, which represents the line of best agreement between the two measures (*r*^2 ^= 0.9526, F = 2372.0, P < 0.0001):

Estimated Haemoglobin = (Haematocrit + 0.6215)/3.1906

When the estimation of haemoglobin using this model was corrected, the new estimated haemoglobin ([Haematocrit + 0.6215]/3.1906) shown that the haemoglobin measurements were lower than corrected observed haemoglobin in 49.2% (59/120), with a linear trend in the relationship significantly more slight than the original (*r*^2 ^= 0.013, F = 1.55, P = 0.216) (Figure 1).

## Discussion

These results showed a consistent bias of haemoglobin measurements to indicate a greater degree of anaemia than haematocrit measurements in the same individuals and populations if the standard threefold conversion is used, as has been reported previously [8], and most recently by Carneiro [1].

The difference between the haemoglobin and centrifuged haematocrit/3 was found to be non-uniform, decreasing with average values of these measures, but conversely not significantly modified by age or sex. These results suggest that the relationship between haemoglobin and centrifuged haematocrit could be independent to the recent exposure to malaria, which is a continuous risk in endemic settings.

Both Hct and Hgb levels could be affected by factors such as the method and equipment used for its determination, environment or subject's differences that may cause a spurious change in the measured value and lead to inaccuracies [2,9].

These and previous data [1,2] have shown that Hgb levels cannot be derived from the Hct values with an acceptable accuracy using the general rule of dividing by 3. The relationship between Hgb and Hct is not exactly 3 and could be affected by age, sex, infection status, malarial etiology and season, among other factors. Due to the lack of agreement, the commonly assumed 'equivalent' cutoff points for anaemia definitions need to be re-evaluated. More information is needed for different aetiologies of anaemia.

## Conclusion

The present study demonstrates that the standard threefold conversion from haematocrit to haemoglobin underestimates the prevalence of anaemia and low levels of haemoglobin in children and adults in a *Plasmodium vivax *malaria endemic settings. In contrast to the results of Carneiro *et al *[1], it was found that the bias was less acute for more severe anaemia defined by haemoglobin<8 g/dl and haemoglobin<5 g/dl.

## Competing interests

The author(s) declare that they have no competing interests.

## Authors' contributions

AJRM analysed and interpreted the data, and wrote the first draft of the paper. CFP designed and co-ordinated the original studies, contributed to data interpretation and to drafting and revising the manuscript. ES, MA, MV, CP and RC were involved in data collection and critically revised the manuscript. All authors read and approved the final manuscript.

## References

[B1] Carneiro IA, Drakeley CJ, Owusu-Agyei S, Mmbando B, Chandramohan D (2007). Haemoglobin and haematocrit: is the threefold conversion valid for assessing anaemia in malaria-endemic settings?. Malar J.

[B2] Quinto L, Aponte JJ, Menendez C, Sacarlal J, Aide P, Espasa M, Mandomando I, Guinovart C, Macete E, Hirt R, Urassa H, Navia MM, Thompson R, Alonso PL (2006). Relationship between haemoglobin and haematocrit in the definition of anaemia. Trop Med Int Health.

[B3] Rodriguez-Morales AJ, Sanchez E, Arria M, Vargas M, Piccolo C, Colina R, Franco-Paredes C (2005). White blood cell counts in *Plasmodium vivax *malaria. J Infect Dis.

[B4] Rodriguez-Morales AJ, Sanchez E, Vargas M, Piccolo C, Colina R, Arria M (2006). Anemia and thrombocytopenia in children with *Plasmodium vivax *malaria. J Trop Pediatr.

[B5] Rodriguez-Morales AJ, Sanchez E, Vargas M, Piccolo C, Colina R, Arria M, Franco-Paredes C (2005). Occurrence of thrombocytopenia in *Plasmodium vivax *malaria. Clin Infect Dis.

[B6] Bland JM, Altman DG (1999). Measuring agreement in method comparison studies. Statistical Methods in Medical Research.

[B7] Bland JM, Altman DG (1986). Statistical methods for assessing agreement between two methods of clinical measurement. Lancet.

[B8] Graitcer PL, Goldsby JB, Nichaman MZ (1981). Hemoglobins and hematocrits: are they equally sensitive in detecting anemias?. Am J Clin Nutr.

[B9] Keen ML (1998). Hemoglobin and hematocrit: an analysis of clinical accuracy. Case study of the anemic patient. ANNA journal/American Nephrology Nurses' Association.

